# A complex four-point method for the evaluation of ohmic and faradaic losses within a redox flow battery single-cell

**DOI:** 10.1016/j.mex.2019.03.007

**Published:** 2019-03-18

**Authors:** P. Mazur, J. Mrlik, J. Charvat, J. Pocedic, J. Vrana, J. Dundalek, J. Kosek

**Affiliations:** a University of West Bohemia, Research Centre – New Technologies, Univerzitni 8, 306 14 Pilsen, Czech Republic; b University of Chemistry and Technology Prague, Dept. of Chemical Engineering, Technicka 5, 166 28 Prague, Czech Republic

**Keywords:** Four-point characterization of flow battery single-cell, Redox flow battery, Electrochemical impedance spectroscopy, Load curve

## Abstract

We propose a complex 4-point method for characterization of flow batteries. The distribution of ohmic and faradaic losses within a single-cell is evaluated from electrochemical impedance spectra and load curves of positive and negative half-cells measured with platinum wire pseudo-reference electrodes positioned in respective electrode compartment. The developed method can be used e.g., for the component screening and *in-situ* durability studies on single-cell scale. The method was validated on a vanadium redox flow battery single-cell; however, it can be analogically employed for various chemistries of flow battery.

• Complex 4-point method for characterization of flow battery single-cell was developed.
• Method is based on electrochemical impedance spectra and load curve measurements.
• Direct evaluation of ohmic and faradaic losses distribution within battery single-cell by the method.

## Graphical abstract

**Figure:**
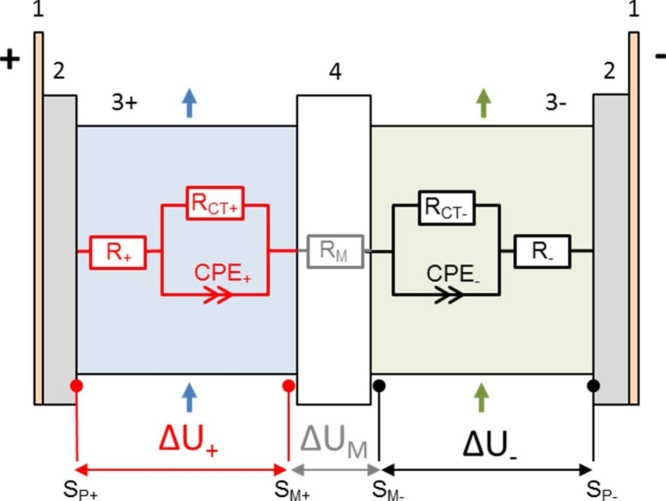

**Specifications Table**

**Table:** 

**Subject area**:	• *Chemistry*
**More specific subject area**:	*Redox flow batteries*
**Method name**:	*Four-point characterization of flow battery single-cell.*
**Name and reference of original method**:	*Standard full-cell characterization of flow battery single-cell.*
**Resource availability**:	*Method can be reproduced with regular materials and chemicals used for flow battery construction and characterization.*

## Method details

We propose a modification of a standard characterization method of flow battery single-cell. Majority of the publications related to research and development of the flow battery component is based on full-cell characterization on a single-cell, typically in symmetric electrode set-up, i.e., same electrode material for cathode and anode [[1], [2], [3], [4]]. However, such approach is not sufficient to precisely evaluate the individual sources of the battery inefficiencies. Due to the flow arrangement, reference electrodes can be easily introduced to the system, typically on the electrolyte inlets which enables to evaluate the polarization of positive and negative electrode independently [5,6]. More recently, the application of dynamic hydrogen electrode composing of platinum wire surrounded between two cation-exchange membranes was used to investigate the inefficiency losses of negative VRFB electrode [7,8]. However, such approach does not provide the information about the distribution of ohmic losses within the real battery single-cell.

**Fig. 1:**
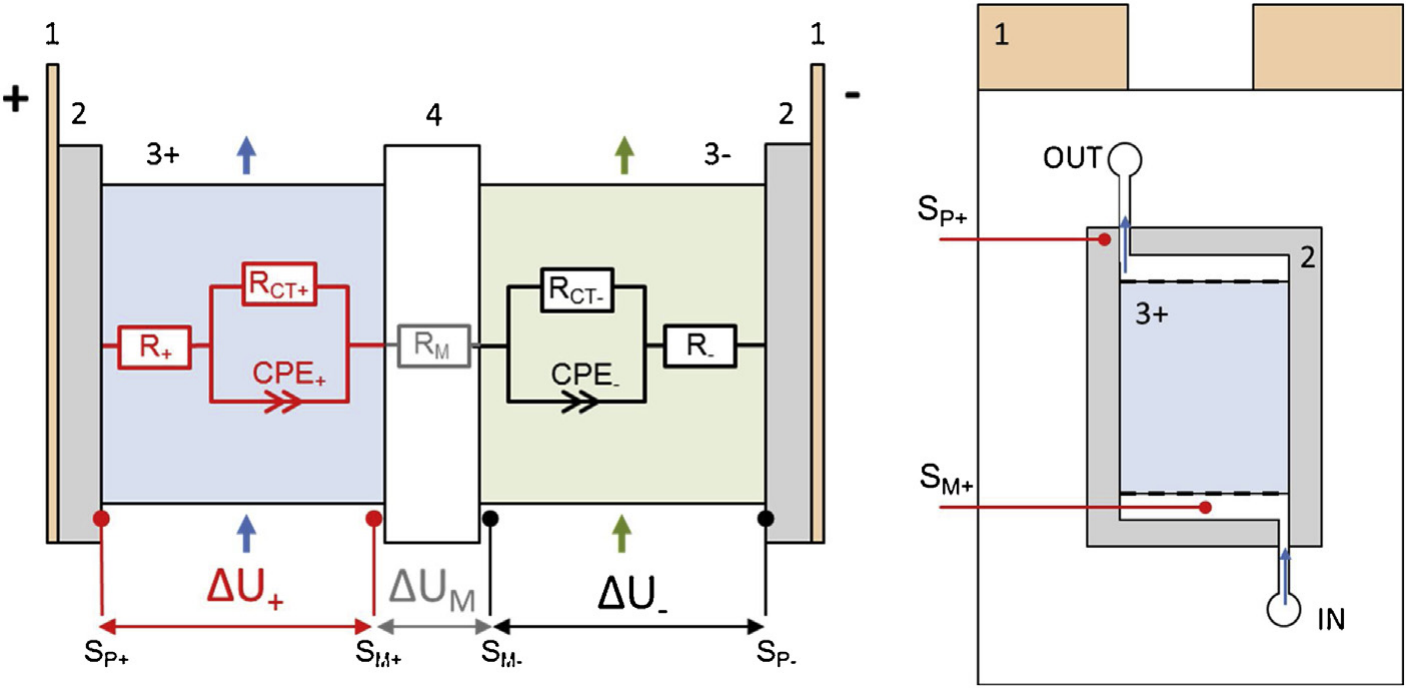
Scheme of VRFB single-cell with indication of positions of platinum wires. 1 – current collectors, 2 – composite plates, 3± – positive/negative felt electrode, 4 – membrane, S_P±_ – platinum wires attached to composite plates, S_M±_ – platinum wires placed in electrolyte inlets surrounding membrane.

**Fig. 2:**
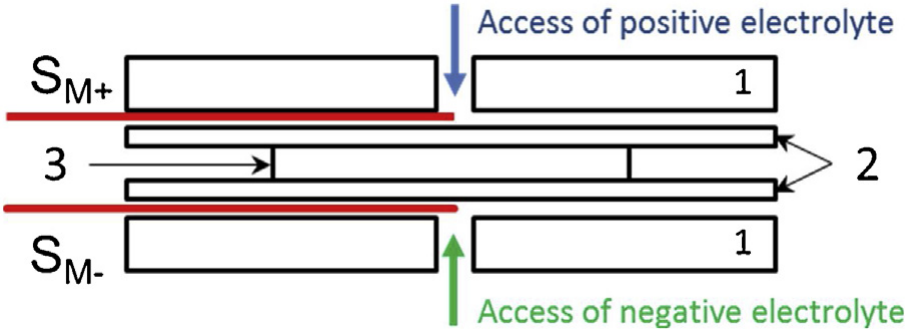
Cross-section scheme of membrane sealings with implemented platinum wire pseudoreference electrodes. 1 – outer membrane sealings, 2 – inner membrane sealings, 3 – membrane, S_M±_ – platinum wires placed in electrolyte inlets surounding membrane.

In our modified method, four high purity platinum wires (0.25 mm diameter) are positioned in the cell according to scheme depicted on Fig. 1. S_M+_ and S_M−_ are placed in the electrolyte inlet channels from both sides of membrane in the proximity of graphite electrodes (without touching them) and they serve as pseudo-reference electrodes [9]. The wires are implemented in the membrane sealing which enables to place the wire close to the membrane, as it is illustrated on cross-sectional scheme on Fig. 2. This was realised by hot-pressing a wire between two thermoplastic elastomeric sheets to fix the position of wire. The defined contact of the wire with inlet electrolyte is realised via a small hole in the outer sealing sheet. The contact of the wire with membrane is prevented by inner sealing sheet which also defines the distance between the wire and membrane to 0.2 mm. During the characterization experiments the changes of the electrolyte composition are negligible, resulting in sufficiently stable potential value of platinum wire.

Platinum wires S_P+_ and S_P-_ are connected to carbon composite plates from the inner side by PTFE adhesive tape (Gore). This enables us to measure the full-cell and half-cell voltage without the contribution of ohmic drop in current collector and bipolar plates.

Such cell arrangement was used for electrochemical impedance spectroscopy (EIS) and load curve (LC) measurements of full-cell and both positive and negative half-cells. From these measurements, the ohmic and faradaic losses of the individual components of the single-cell can be evaluated. The following experimental specifications of the characterizations were used:

• EIS with 5 mV potential amplitude in the frequency range from 200 kHz to 50 mHz. The obtained EIS spectra were fitted to appropriate equivalent circuit using EC-Lab software tool.
• LC measurements with linearly increasing current scan (from 0 to +200 mA cm^−2^ for charging and from 0 to – 200 mA cm^−2^ for discharging) with 1 mA cm^−2^ s^−1^ scan rate. The slopes of charge and discharge U-j curves of full-cell and both half-cells were taken as the value of area-specific resistance under load.

Battery testing was carried out with Potentiostat/galvanostat/FRA EC-LAB VSP-126 (Biologic) with 4 A current booster. All the measurements were performed at 20 °C using tempered box.

## Method validation

The method was validated using a vanadium redox flow battery single-cell of our own design and construction. The battery body was manufactured from polyvinylchloride and sealed by elastomeric gaskets. The graphite felt electrodes (each of 20 cm^2^ active area and 25% compression of the original thickness) were supported by carbon composite plates PPG86 (Eisenhuth). The electrode compartments were mutually separated by PFSA type cation-exchange membrane (Fumatech). All the measurements were conducted under electrolyte flow rate of 40 cm^3^ min^−1^, which corresponds to the linear flow velocity of approx. 27 cm min^−1^. Commercial electrolyte comprising 1.6 mol dm^−3^ of vanadium ions (with 1:1 M ratio of V^3+^:V^4+^, 2 mol dm^-3^ of H_2_SO_4_ and 0.3% of H_3_PO_4_ (GfE, Gesellschaft für Elektrometallurgie mbH) was used as the initial catholyte and anolyte. Nitrogen gas was bubbled through the negative electrolyte to prevent its self-discharge due to the oxidation of V(II) ions by oxygen from air.

**Fig. 3:**
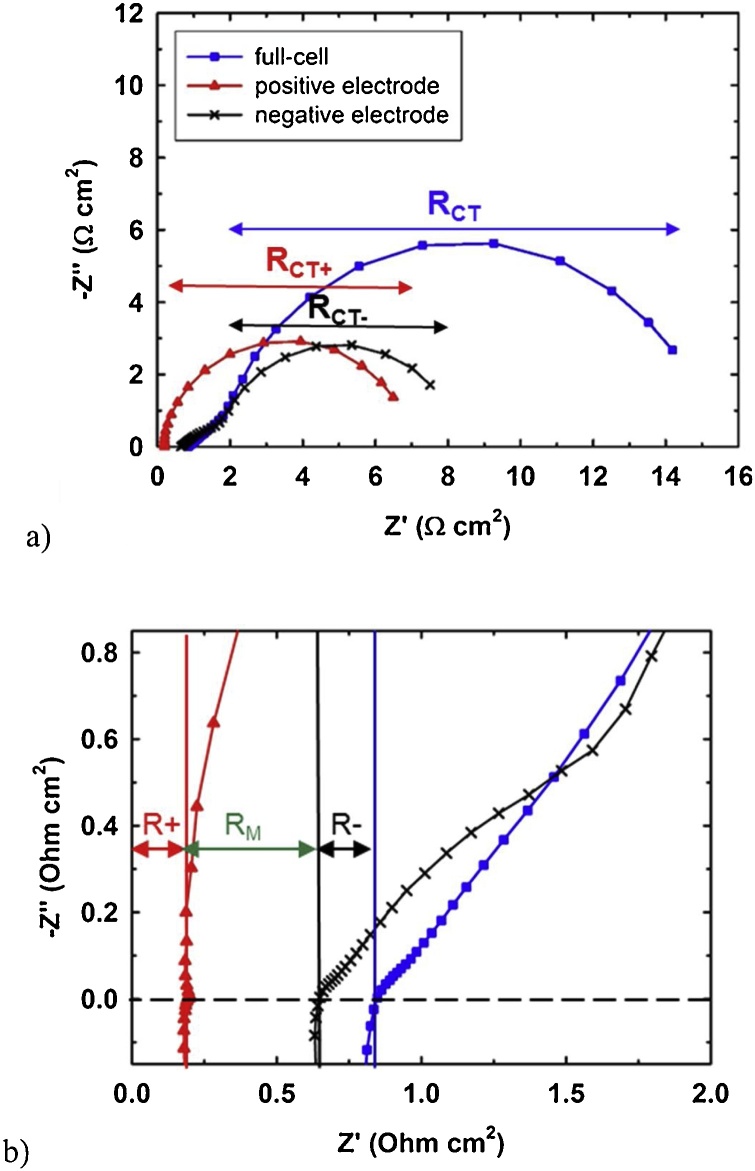
EIS spectra of vanadium redox flow battery single-cell in symmetric electrode set-up: full-cell (blue squares) and both positive (red triangles) and negative (black crosses) half-cells measured with S_M+_ in –50% SOC electrolytes. Full spectra (a) and zoom of high-frequency region (b) with indication of distributions of ohmic resistances within the cell.

**Fig. 4:**
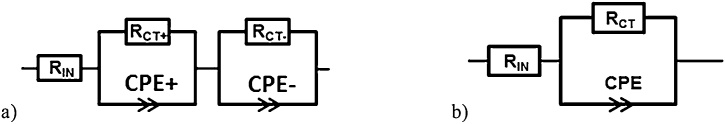
Equivalent circuit used for the evaluation of full-cell (a) and half-cell (b) EIS spectra.

The battery was characterized in two different state-of charges (SOC). Firstly, characterization was performed in initial electrolytes, i.e., in equimolar mixture of V^3+^ and V^4+^ in both tanks. The resulting EIS spectra of single-cell and positive and negative half-cells are presented in the form of Nyquist diagrams on Fig. 3a, with zoom of high-frequency region on Fig. 3b. The equivalent circuits shown on Fig. 4 were used to fit the spectra.

According to the theory [7,10,11], the intercept of the curve with horizontal axis represents the ohmic resistance of the cell ($R_{IN}$) and the diameter of the flattened semi-circle in low frequency region represents the charge transfer resistance ($R_{CT}$) of electrode reactions. The flattening of the semi-circle is caused by non-uniform potential distribution within porous graphite felt electrode and thus the constant phase element (CPE) was used in equivalent circuit instead of capacitor to account for unideal behaviour of electrode-electrolyte double layer capacitance. EIS spectra of the full-cell contains only a single semi-circle in low frequency region corresponding to both positive and negative electrode-electrolyte interfaces, which are identical (same electrodes and same initial electrolyte composition). In fact, $R_{CT}$ composes of two identical overlapped semi-circles of positive ($R_{CT+}$) and negative ($R_{CT-}$) electrode reactions, which were obtained by EIS characterization of respective half-cells.

**Fig. 5:**
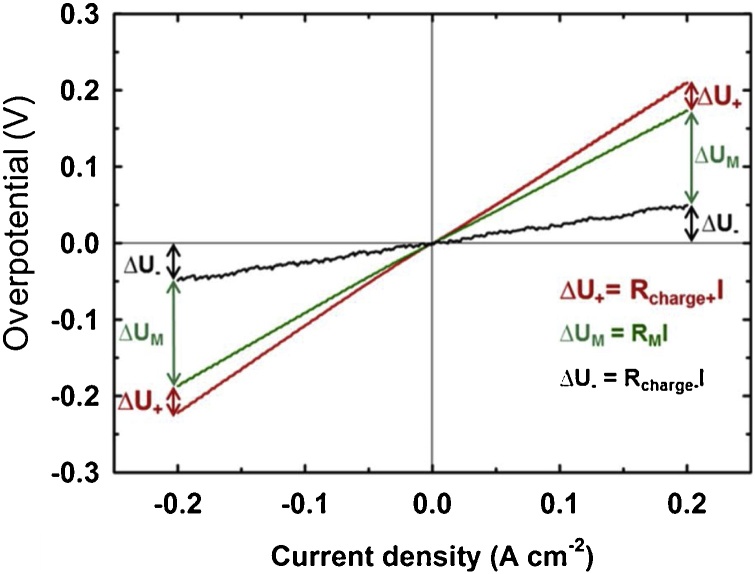
Load curves of vanadium redox flow battery single-cell in symmetric electrode set-up with S_M+_ and S_M–_ in +50% SOC electrolytes.

From the high-frequency region of the spectra we can evaluate the distribution of ohmic resistances within the single-cell, as it is indicated on Fig. 3b. For better visualization, we present both half-cells spectra obtained with positive pseudo-reference electrode (S_M+_). Subsequently, the battery was charged by applying constant current to +50% (SOC) (defined by equimolar ratios of V^4+^:V^5+^ and V^2+^:V^3+^ in catholyte and anolyte, respectively) and the EIS characterization was repeated, together with LC measurements. On Fig. 5 the full-cell and half-cell LCs are compared. The overpotential values were calculated by subtracting open circuit potential from the potentials under current load. We observed linear dependence of overpotential on current density in a broad range of current density of 0–200 mA cm^−2^. Thus, the resistances of individual cell components under current load can be calculated from the slopes of the curves both for charging and discharging.

The data evaluated from the EIS characterization in –50% SOC and +50% SOC are summarised in Table 1. In the last table row, the resistances of individual components (evaluated from half-cell measurements) are summed-up and good accordance with full-cell values is documented. The results obtained by EIS correlates well with the results of LC measurements. From these data we can see that, for the measured single-cell, the efficiency losses originate mainly from ohmic resistance of membrane and both electrodes. Slightly higher activation polarization is observed for negative electrode.

**Table 1:** Summarization of the distribution of ohmic and faradaic resistances within the single-cell obtained from EIS characterization in –50% SOC and +50% SOC.

	–50% EIS		+50% EIS		+50% LC	
	R_IN_	R_CT_	R_IN_	R_CT_	R_charge_	R_discharge_
	Ohm cm^2^	Ohm cm^2^	Ohm cm^2^	Ohm cm^2^	Ohm cm^2^	Ohm cm^2^
Full-cell	0.85	12.80	0.87	0.20	1.06	1.12
Positive electrode	0.21	5.68	0.22	0.08	0.29	0.28
Negative electrode	0.20	6.74	0.20	0.13	0.35	0.34
Membrane	0.44	–	0.45	–	0.45	0.49
SUMMARY	0.85	12.42	0.87	0.21	1.09	1.11

We demonstrated that the distribution of ohmic and faradaic losses within single-cell can be evaluate from EIS and LC measurements in full-cell and half-cell arrangement using Pt wire probes inserted in the cell. The developed four-point characterization method can be used for various investigations of redox flow battery single-cell scale, e.g., focused on component performance and durability. The risk of hydrogen evolution reaction of negative electrolyte at higher SOC catalysed by Pt wire pseudo-reference electrodes was taken into account. By comparing single-cell parameters of the same system with and without Pt wires, we have not observed any difference on coulombic efficiency and battery capacity (data are not shown here). Thus this effect seems to be negligible for our system, probably due to very small area of Pt exposed to the environment (only tip of wire of low surface roughness). But in general, Pt can be easily substitute by other chemically stable electron conductive material, e.g., carbon fibre, if needed.
